# HIV is Down, but Not Out Yet

**DOI:** 10.4103/0970-0218.62545

**Published:** 2010-01

**Authors:** Rajesh Kumar

**Affiliations:** PGIMER School of Public Health, Chandigarh - 160 012, India

I am grateful to IAPSM for inviting me to deliver this prestigious oration. All of you are familiar with the life and work of Dr. Harcharan Singh, who was a great teacher of Preventive and Social Medicine. He also made significant contributions to Indian public health while working with the Planning Commission of India. I have heard him speak only once and that was when he came to my institution to address the faculty. He was a very good orator. I still remember him emphasizing the role of knowledge in changing attitudes and behaviors. That, to him, was the key approach in public health.

I would like to elaborate on how this key public health approach has helped in controlling HIV in India as well as in several other countries. HIV appeared as a new disease in the early 1980s. In 1986, it was identified among some sex workers of Chennai. By the 1990s it had spread to every nook and corner of India, threatening the socioeconomic stability of the region. HIV posed a formidable challenge to public health. The use of the traditional public health tools of epidemiological surveillance made it possible to identify vulnerable population groups and geographical areas. AIDS case surveillance provided valuable information. The heterosexual transmission route, and especially sex work, was identified as the major mode of transmission in this country and this paved the way for the launch of large-scale preventive efforts.

HIV spread rapidly in many countries of Africa in a short period of time. The first victims were sex workers; from them it spread to their clients and, finally, to the wives of these clients. Going by the experience in Africa, it was predicted that by 2015 there would be about 5-10 million cases of HIV in India. However, there were a few people who disagreed with these figures; notable among them was Dr. N. S. Deodhar, former Director of All India Institute of Hygiene and Public Health, Kolkata, who predicted that HIV would never affect large populations in India as sexually transmitted infections (STIs) never had a high incidence in this country. However, despite limited information, the epidemiological projections provided the basis for advocacy that generated unprecedented political will and financial resources.

The need for launching organized social action, the cornerstone of public health approach, led to the establishment of the National AIDS Control Organization (NACO) in India and UNAIDS at the United Nations for coordinating the actions not only in health ministries but in all relevant sectors. As there was no drug or vaccine that could prevent or treat the infection, prevention by behavior change, a key public health approach, occupied central position in the National AIDS Control Program. Behavior change interventions are still a major focus in this program. Adoption of the health promotion approach provided an enabling environment to communities for behavior change; this was backed by supportive policies recognizing human rights as a core value.

The sentinel surveillance system which came into being in the late 1990s provided an excellent opportunity for tracking not only AIDS-related deaths, but also HIV infection and sexual behaviors in different population groups and geographic areas. Multiple sources of information–e.g., cause-specific mortality rates from the sample registration system (SRS), sentinel surveillance of HIV infection and sexual behaviors, and household surveys in representative populations–have helped in constructing a realistic picture of the epidemic in India. The key question, however, is whether there is a change in HIV or STI incidence. Can the change be explained by the presence of some unknown bias or confounder? And if there is a change, what is causing the change? Is the change in behavior due to mass media campaigns that are directed at the general population or due to the targeting of the behavior change interventions to high-risk groups? Biological and behavioral surveillance should be able to provide the answers to these questions.

Epidemiological analysis of HIV sentinel surveillance provided the first indication of the declining trend in HIV prevalence among young pregnant women in some of the Southern states. A similar trend was observed among young male STI clinic attendees. This observation was shared with the public health community by a publication in the Lancet in 2006. However, eminent public health physicians and epidemiologists from leading institutions of India attributed the observed declines to various biases or confounders. Poor coverage of antenatal clinics in northern India, expansion of the surveillance to rural areas, and nonrepresentation of older women were proposed as alternate explanations. This constructive criticism led to further analysis, which included analysis of surveillance data up to 2007; the results confirmed the earlier observation. However, the search for bias and confounding continues in the true spirit of epidemiological inquiry.

Stratified analysis revealed similar HIV declines when analysis was restricted to sites that were consistently sampled throughout the last 8 years. Illiterates and rural residents showed a similar trend. HIV decline was also seen among male STI clinic attendees. Syphilis prevalence among young pregnant women and young men attending STI clinics also showed a similar trend. The decline in HIV and STIs seems to be real and cannot be explained by bias or confounding or change in the characteristics of host or agent. The most likely reason for the decline is a change in the sexual behavior of key population groups, i.e., sex workers and their clients. Analysis of causes of death from 2001-2003 by verbal autopsy and the third National Family Health Survey also support the low HIV prevalence estimates in India.

Behavior surveillance surveys provide valuable information. It is well known that due to the social desirability bias in face-to-face interviews, females tend to underreport sex with a male other than their regular partner and, similarly, males also underreport having sex with another male. Nevertheless, trends in sexual behaviors can be assessed using less-than-perfect interview methods. Analysis of behavior surveillance surveys for the high- and low-HIV-prevalence states indicate that the prevalence of multi-partner sex has declined to some extent and safe sex practices have improved in the high- as well as low-prevalence states. This is also corroborated by sex behavior surveys of female sex workers and their clients. The number of sexual partners has declined in the general population for both males and females, though those in the upper tail of the distribution may not have changed that much. Similarly, the number of paying sex partners of female sex workers shows a decline. The clients of female sex workers have also reported a decline in the number of paid sex partners though, again, the change is less pronounced in the upper end of the distribution. This indicates that a small minority still engages in unsafe sexual behavior, which still provides a niche for propagation of HIV. Overall, safe sexual practices have become a norm in both high- as well as low-HIV prevalence states. A decline in the prevalence of HIV has also been noted among female sex workers. Small cross-sectional and cohort studies in Kolkata and Pune, respectively, confirm this trend. However, the epidemic continues to rage among men having sex with men and in injecting drug users–the risk groups that were neglected in the past.

Knowledge of local HIV epidemiology is essential for choosing the appropriate response. HIV epidemiology varies a lot in the country, not only between states but also between districts and even within the district. The size of HIV sentinel surveillance data at the district level is not sufficiently large to guide local action; hence, program data from the Integrated Counseling and Testing Centers from STI patients, pregnant women, voluntary blood donors, and antiretroviral therapy (ART) clinic attendees should be analyzed. Recently, we found that HIV prevalence from the Prevention of Parent to Child Transmission (PPTCT) sites is not significantly different from that of sentinel surveillance sites. PPTCT data is of sufficient size to measure trends in smaller geographic areas and population groups provided biases are carefully evaluated.

Essentially, in India the HIV epidemic remains concentrated in high-risk groups such as injecting drug users and male and female sex workers. To contain the epidemic, it is essential to map high-risk groups so as to target preventive interventions using the health promotion strategy. So far, the response to the HIV epidemic had been a population-wide biomedical-oriented preventive approach and this has paid off. However, to root out the causes of HIV spread, the social determinants need to be tackled.

No one knows better than us that the origin of disease lies in the socioeconomic conditions in which people live and work; therefore, social structures and systems need to be challenged if we are to have policies that favor health and wellbeing. A society that generates a large number of single male migrant workers will give rise to a demand for sex work. Therefore, social action is needed to create a just and humane social system that will generate less disease.

The health promotion approach as described in the Ottawa Charter combines health education and healthy public policies. Health is not only an individual responsibility but also a social one. Asking people to change without, at the same time, bringing about a change in social policies, is not ethical. Success depends on individual and community empowerment. Building healthy policies requires creation of a supportive environment for community action, including development of skills for behavior change. Public health professionals need to acquire skills for advocating changes in the policies that support behavior change by making healthy choices the easier choices. Rudolf Virchow, the father of social medicine, once said ‘epidemics are a reflection of the disturbances in the social system,’ ‘medicine is a social science,’ and ‘politics is nothing but medicine on large scale.’ This explains the importance of the work that needs to be done at the policy level; unfortunately, at present, it is not getting due emphasis in our work.

HIV is down, but not yet out. All those who are infected will continue to need care for a number of years. According to current estimates about 25 lakh people are infected with HIV and about 2.2 lakh are on ART. As the number of persons needing treatment increase in the near future, it will be difficult to run the testing and treatment service as a vertical activity; hence, steps must be initiated to integrate care and support activities within the general health services. For sustaining the gains achieved so far, the AIDS control program should focus its attention on the social determinants of HIV, while continuing to target primary prevention in vulnerable communities and geographic areas.

